# Do Nonsteroidal Anti-Inflammatory Drugs Affect Bone Healing? A Critical Analysis

**DOI:** 10.1100/2012/606404

**Published:** 2012-01-04

**Authors:** Ippokratis Pountos, Theodora Georgouli, Giorgio M. Calori, Peter V. Giannoudis

**Affiliations:** ^1^Academic Department of Trauma & Orthopaedics, School of Medicine, University of Leeds, Leeds LS1 3EX, UK; ^2^Academic Department of Trauma & Orthopaedics, School of Medicine, University of Milan, 20122 Milano, Italy; ^3^Academic Unit, Department of Trauma and Orthopaedics, Clarendon Wing, Leeds Teaching Hospitals NHS Trust, Great George Street, Leeds LS1 3EX, UK

## Abstract

Nonsteroidal anti-inflammatory drugs (NSAIDs) play an essential part in our approach to control pain in the posttraumatic setting. Over the last decades, several studies suggested that NSAIDs interfere with bone healing while others contradict these findings. Although their analgesic potency is well proven, clinicians remain puzzled over the potential safety issues. We have systematically reviewed the available literature, analyzing and presenting the available *in vitro* animal and clinical studies on this field. Our comprehensive review reveals the great diversity of the presented data in all groups of studies. Animal and *in vitro* studies present so conflicting data that even studies with identical parameters have opposing results. Basic science research defining the exact mechanism with which NSAIDs could interfere with bone cells and also the conduction of well-randomized prospective clinical trials are warranted. In the absence of robust clinical or scientific evidence, clinicians should treat NSAIDs as a risk factor for bone healing impairment, and their administration should be avoided in high-risk patients.

## 1. Introduction

### 1.1. Bone Healing

**Figure 1:**
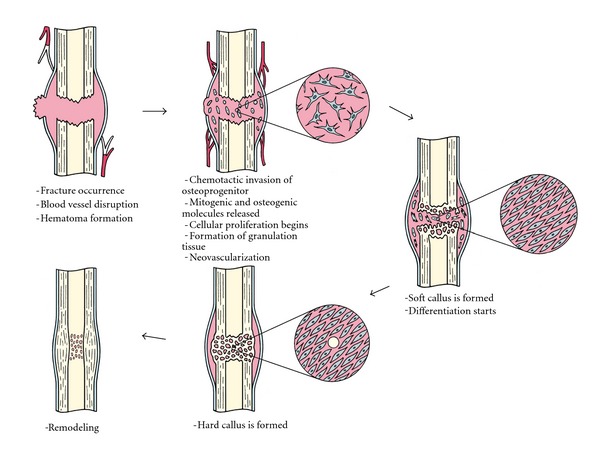
The fracture healing cascade.

Bone healing is one of the most complex cascades of events aiming to the repair of fractured bone without the formation of scar tissue [1]. In this physiological process, several cell types participate along with signal pathways and alternations in the biochemical profile of the local area. Bone healing can be either primary (direct) or secondary (indirect), [1, 2] with the majority of fractures heal indirectly, that is, a process subdivided in several stages [1]. The indirect fracture healing begins immediately after fracture occurrence with the disruption of local blood supply, hypoxia, and the formation of a hematoma,** (** Figure 1) [1]. Cytokines and growth factors are released both locally and systemically and induce a mitogenic and osteogenic effect on the osteoprogenitor cells [3–5]. The formation of new blood vessels, in association with further growth factor and prostaglandin production, promotes differentiation of mesenchymal stem cells (MSCs) toward chondrogenic or osteogenic lineages, forming initially woven bone and in turn, the hard callus [3, 6–8]. Finally, this process is followed by an extended period of remodeling characterized by resorption and new bone formation, resulting in the restoration of mechanical strength and stability [8].

### 1.2. Factors Affecting Bone Healing

The outcome of bone healing can be affected by a diversity of local and systemic factors with varying degrees of affection including fracture gap and comminution, disturbances of blood flow, degree of soft tissue damage [9, 10], insufficient mechanical stability, [10–13], poor nutritional state, age, and smoking [14, 15]. Another important factor that can interfere with the body's ability to heal a fracture is the administration of several pharmacological agents [15]. Steroids, chemotherapy drugs, and some classes of antibiotics have been reported to exert a negative effect on bone healing[15, 16]. In addition, NSAIDs that are one of the most commonly prescribed drugs for pain relief and inflammation to date have also been found to delay union and to inhibit fracture healing [15].

### 1.3. NSAIDs Physiology

Nonsteroidal anti-Inflammatory drugs (NSAIDs) have their origin in the extracts of salicylate-containing plants initially described in ancient Roman and Greek literature, with the willow tree extract to be renowned for their antipyretic, analgesic, and anti-inflammatory properties [17]. Their mode of action remained unknown till the 1970s when Sir John Vane demonstrated the inhibition of the enzymatic production of prostaglandins by NSAIDs [5].

During the biosynthesis of prostaglandins, cyclo-oxygenase (COX or prostaglandin H synthease) catalyses the conversion of arachidonic acid to the prostaglandin endoperoxidases PGG_2_ and then PGH_2_ [18, 19]. PGH_2_ is the precursor for the biological active prostaglandins and thromboxanes. PGH_2_ is then isomerized into various prostanoids such us thromboxane A_2_ (TXA_2_), prostacyclin (PGI_2_), PGD_2_, PGE_2_, and PGF_2a_ [18, 19].

However, COX-1 is constitutively expressed in the most of cells and is involved in physiological processes. In the gastrointestinal tract (GI), prostacyclin and PGE_2_ exert a protective effect by reducing acid secretion, vasodilatation of blood vessels of gastric mucosa, and stimulation of production of mucus which acts as a barrier [20]. In the kidneys, prostaglandins play a key role in regulating blood flow and enhancing organ perfusion [20]. COX-1 expression is also found in faetal and amniotic cells, uterine epithelium in early pregnancy, and central nervous system, and it is thought to exert complex integrative functions [21]. COX-2, on the other hand, is considered to be induced by inflammation and by the presence of proinflammatory cytokines and mitogens [22]. It has been suggested that the anti-inflammatory action of NSAIDs is due to the inhibition of COX-2, whereas COX-1 inhibition is associated to unwanted effects related to interference of the regulatory and protective mechanisms [22, 23]. Recent studies, however, have indicated that COX-2 is also constitutively expressed in the brain, and in particular, in the hippocampus and cortical glutaminergic neurons as well as the kidneys, uterus, and prostate [24, 25]. Similarly, COX-1, despite its constitutively expression, has been shown to participate in inflammation (e.g., lipopolysaccharide-induced inflammation) where it might be inducible [26].

### 1.4. Prostaglandins during Fracture Healing

Prostaglandins (PGs) are autocrine and paracrine lipid mediators produced by several cell types capable of mediating either a stimulatory or resoptive role depending on the physiological or pathological conditions [27]. Administration of prostaglandins in animal models has shown to increase cortical and trabecular mass and cause hyperostosis in infants [28, 29]. Similarly, local administration of PGs in rat long bones had stimulatory properties suggesting direct effect on bone by inducing osteogenesis [30]. At a cellular level, PGs have a direct effect on osteoclasts leading to increased bone resorption by a mitogenic effect and increasing their functional activity [31]. On the other hand, PGs can exert an anabolic effect on the bone by increasing the multiplication and differentiation of osteoblasts [32]. One could claim that PGs safeguard the balance between bone resorption and bone formation [33].

Following a fracture, local release of PGs occurs early as a result of the acute aseptic inflammatory response [34]. COX-2 plays a critical role in this phase and its induction in osteoblasts is essential for bone healing [35]. In COX-2 null mice, fracture healing was found impaired characterized by reduced bone formation and persistence of mesenchyme and cartilage [36]. In the same study, COX-1 knockout animal was found to have the same healing potential to that of the normal wild type [36]. COX-2 activation therefore is a local regulator of cellular response within bone and responsible for the production of PGs [37]. It is not yet clear what the exact mechanism of PGs on bone cells is; however, it was found that PGE2 regulates BMP-2, BMP-7, and RANKL expression [38–40], and it can increase cell numbers through suppression of apoptosis without direct effect on proliferation [41, 42]. PGs exert this range of action through a variety of receptors expressed. These receptors belong to the G-protein-coupled receptor family and are the EP1, EP2, EP3, and EP4 subtypes [40]. Although the role of each receptor is not fully explored, studies suggest that the PGE2 binding to EP4 can stimulate osteoclastogenesis and osteoblastic differentiation, and animal models lacking the EP2 and EP4 receptors had defects in bone metabolism [43]. On the contrary, EP1 null mice found to have accelerated fracture repair and MSCs isolated from their bone marrow had higher osteoblast differentiation capacity and accelerated bone nodule formation and mineralization *in vitro* [44]. 

### 1.5. NSAIDs and Analgesia

In acute pain after fracture or during the postoperative period after fracture fixation, NSAIDs play an important role due to their pronounced analgesic potency, anti-inflammatory effects, and lesser side effects compared to opioids [7, 45]. However, studies comparing opiates and NSAIDs have shown that NSAIDs are at least as effective as opiates with some studies suggesting that NSAIDs can achieve greater reductions in pain scores [46–52]. For acute pain, it has been suggested that NSAIDs should be used as the first line of treatment in pain therapy and recommend that opioids should be added only if pain is not controlled adequately with NSAIDs alone [50]. Furthermore, the use of NSAIDs instead of narcotic analgesics avoids significant side effects like respiratory depression, sedation, and cognitive effects [53]. For the postoperative patients, this can be translated with decreased hospital stay, allowing early mobilization and weight bearing [15, 46, 51, 52].

While there are clear benefits supporting the administration of NSAID's as pain relief agents following fractures, their wide-spread use has been challenged due to their reported negative impact on the bone repair processes [47, 51, 52]. Do NSAIDs inhibit the healing of fractures? Can they safely be administered?Ifso, at what time point and for how long? In order to provide replies to the above queries, we undertook a comprehensive review of the literature.

## 2. Materials and Methods

We searched available literature through PubMed, OVID, and EMbase with general keywords including “mesenchymal stem cells (MSC's),” “Bone healing,” and “Bone marrow-derived stem cells” both isolated or in combination with specific words including generic words like “NSAIDs” or specific names of NSAID's from January 1980 to January 2011. For paper selection, the initial inclusion criteria were studies publishing results on the effect of NSAIDs on bone healing *in vivo* both in humans and animal models, and also *in-vitro* studies on the effect of these agents on osteoprogenitor cells. Exclusion criteria included publications in languages other than English or studies with unclear methodology [54, 55]. The papers describing the effect of NSAIDs on bone healing were reviewed and are presented below.

## 3. Results

**Figure 2:**
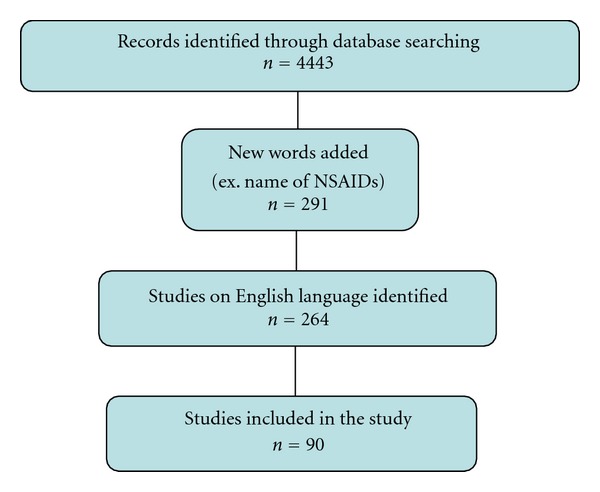
Flow chart diagram of included studies.

Out of 4443 papers that were initially isolated, 90 meet the inclusion criteria (Figure 2) [56–145]. Studies selected were grouped as experimental (*in vivo* or *in vitro*) or clinical as described below.

### 3.1. *In-Vitro* Models

We identified 18 *in-vitro* studies analyzing the effect of NSAIDs on osteoblasts and MSCs ability to proliferate and differentiate toward osteogenic lineages, (Table 1) [56–73]. An early study, byTörnkvistet al., using mesenchymal limb-bud cells reported no effect on osteogenesis and chondrogenesis by indomethacin [56]. However, the latter studies presented a diversity of results. The proliferation potential of osteogenic cells was found inhibited, and the higher the concentration, the more potent the antiproliferative effect was [57, 58, 60, 68, 71]. Interestingly, replenishment of PGE-1, PGE-2 and PGF2a did not reverse this negative effect [64, 68]. Other studies showed no effect on low concentration and reported a negative effect at higher ones [63, 67, 69, 72, 73].

The osteogenic potential of the studied cells, measured by the levels of ALP activity and calcium production, was either found increased or unaffected in the majority of the studies [56–59, 62, 73]. NSAIDs also reported to stimulate collagen synthesis [57, 58]. On the contrary, other researchers failed to reproduce this result showing disruption of osteogenesis [65, 72]. Kellinsalmi et al. reported that indomethacin, parecoxib, and NS398 inhibited osteoblastic differentiation of human MSCs and found a significant increase of adipocytes suggesting diversion to adipogenesis instead of osteogenesis [65].

 In terms of chondrogenesis, limited studies exist which either present no effect [72] or a negative effect in expected concentrations after administration [73]. In an attempt to explain this wide diversity of results, it was apparent that cells were isolated from a variety of species and sites [56–58, 66, 68, 69, 72, 73]. However, we could not find any association between them, and no association was apparent between different NSAIDs or even selectivity toward the COX-1 or COX-2 enzyme to explain these results.

### 3.2. Animal Models

A large volume of work has been undertaken over the last 4 decades using experimental fracture animal models. The majority of these studies were centred over rodents or rabbits, and as with the *in-vitro* studies, great diversity and controversial results have been presented in the 54 studies identified (Table 2) [74–127]. A proportion of these studies suggest that NSAIDs adversely affect the bone physiology by delaying bone healing and callus formation, impairing bending stiffness and the bones' mechanical properties leading to an increased rate of nonunions [74–105]. Some authors have even compared NSAIDs effect on fracture healing with that of other pharmacological agents like steroids [89, 91]. Høgevold et al. presented that short-term administration of indomethacin inhibits fracture healing while this was not the case with short-term administration of methylprednisolone [89].

On the other hand, several studies failed to reproduce this effect suggesting that NSAIDs have no effect of fracture healing [110–125]. The results were so controversial that different researchers with identical animal fracture models, same drugs, and same doses presented opposite outcomes [122, 127]. Analysing these studies further, we could not identify any association among the class or potency of the studied NSAIDs to inhibit the COX-1 or COX-2 enzyme, the dose, or the timing. In terms of timing, although some authors suggest that short-term administration after fracture could be safe, others contradict this finding suggesting that NSAID administration is safe only if it is initiated a few weeks after fracture [78, 85, 98, 101, 105, 106, 108, 126]. A link, however, can exist between the size of the animals. The vast majority of the animal models involved rodents or rabbits. There are two studies that involved dogs and goats whose results showed no inhibition of bone healing or bone ingrowth suggesting that the type or size of the animal model used might be an explanation for the differences seen in the results presented [113, 117].

### 3.3. Clinical Studies

There are only a few retrospective human studies and even fewer prospective randomized trials studying the effect of NSAIDs after fracture or spinal fusion, (Tables 3 and 4) [128–145]. In a double-blinded randomized control trial, Adolphson et al. found that piroxicam had no effect on the healing potential of 42 postmenopausal women with displaced Colles' fractures [137]. Similar findings were reported by Davis and Ackroyd who studied the effect of fluriprophen on Colles' fracture healing potential [136]. In cementless hip arthroplasty, indomethacin was found to have no effect on the prosthetic loosening. No effect was also reported in a randomized, controlled, and blinded study by Sculean et al. who studied the effect of rofecoxib on the healing of intrabony periodontal defects [143]. On the contrary, four retrospective studies suggested that patients using NSAIDs after fracture had a higher incidence of nonunion compared to those that did not [140–142, 144]. Bhattacharyya et al., have suggested that patients receiving NSAIDs within 90 days after fracture had a 3.7-fold risk for nonunion, while the risk for opioids users was 1.6 folds [144].

Detrimental effects in spinal fusion are presented by some authors, while others concluded that NSAIDs do not affect union. Park et al. found that the incidence of incomplete union or nonunion was much higher in patients taking ketorolac and the relative risk was approximately 6 times higher compared to that of the control group [131]. A more recent study by Lumawig et al. indicated that diclofenac sodium showed a dose-dependent inhibitory effect toward spinal fusion especially when used during the immediate postoperative period [134]. In addition, it was pointed out that patients who continued to take NSAIDs for more than 3 months postoperatively showed significantly lower fusion and success rates [128]. On the contrary, other studies failed to support these findings suggesting that NSAIDs do not affect union after spinal fusion [130, 133, 135].

## 4. Discussion

For many years, NSAIDs have played an essential part in our approach to control pain in the posttraumatic setting. However, several authors highlighted that NSAIDs could inhibit the bone healing process. The available data from animal studies have all evaluated the properties of newly formed bone in animals that NSAIDs were administered in different doses and durations. One could expect some uniformity in these results but on the contrary great diversity and conflicting results exist. It is of question how so many studies have failed to provide a clear message with regards to the effect and mode of action of NSAIDs on bone healing in animals. The differences reported were not only between species, dose, and duration of administration but also between identical parameters. For instance, 30 mg/kg/day of ibuprofen given orally in rats had no effect on femoral fracture by Huo et al. [122], but retardation of fracture healing with significant differences of mechanical and histological properties was reported by Altman et al. [127]. Many researchers have also chosen long-term administration of NSAIDs or high doses that is against the intended human therapies. For example, Leonelli et al. compared the effect of 30 mg/kg/day of Ibuprofen and 8 mg/kg/day of rofecoxib on the union potential of a closed femoral fracture in a rat model [94]. Their results showed nonunions in 64.7% of rofecoxib-treated rats and 17.6% of ibuprofen-treated rats but the dose of rofecoxib used was more than 10 times the dose given in humans for acute pain. It is also unclear whether this diversity of results is due to inter- or intraspecies differences, compensatory local and systemic factors, or even different pharmacokinetics of the drugs in the laboratory models compared to humans. It is possible that secondary factors influence the final result like the level of analgesia achieved by a specific dose and class of NSAID affecting the weight bearing status for some animals and thus to evolution of healing.

The extent of trauma and the comminution of the fractures produced in these experimental models, we believe, is another major factor that could explain the differences between researchers. Differences in the fracture comminution, force used, soft tissue damage, and fracture stability achieved can all influence the final result. This argument can be further strengthened by the observations of Engebretsen et al., who reported that indomethacin exerted a negative effect in unstable fracture condition while in stable ones had no significant effect compared to controls [78]. This could also explain the high complication rates presented even for the control animal groups. Long et al., for instance, studied the effect of celecoxib and indomethacin on a rabbit model of spinal fusion [119]. Their results showed fusion rates of 64% in the control group, 45% for the celecoxib group, and 18% for the indomethacin group. Although a significant negative effect is presented with these two NSAIDs, a nonunion rate of 36% is high to be used as baseline. It is possible, for example, that NSAIDs interfere only in endochondral ossification, therefore, if the fracture is comminuted and highly unstable, NSAIDs might have a detrimental effect on the consolidation process, while in a more stable fracture, NSAIDs could be totally ineffective.

One positive association could be the fact that the majority of the animal studies involved small animals, that is, rodents and rabbits, while the available studies involving goats and dogs show no effect of bone ingrowth or healing [113, 117]. This could be an important finding as marked interspecies differences with regard to bone composition, bone density, bone mechanical competence and bone cells exist and are more pronounced in small rodents while dogs approximate human bone properties the best [146]. On the other hand, NSAIDs pharmacokinetics could be significantly different between species and humans, and in fact studies analyzing NSAIDs kinetic parameters show significant differences [147, 148]. Despite the above-mentioned limitations of animal models, further studies will be needed to strengthen these assumptions.

The *in-vitro* studies follow exactly the same pathway, being inconclusive and difficult to interpret. Some studies present a strong effect of NSAIDs on the potential of osteoprogenitor cells to proliferate and differentiate toward an osteogenic lineage while others refute it (Table 1). A determining factor could be the use of cells from a variety of species which include cells from rodents, chicken, horses, dogs, and pigs as well as human cells [56, 73]. This diversity of cell sources could be crucial as cells from animals could react in a different fashion to the human cells and in fact according to our experience, animal MSCs exert different proliferation and differentiation rates compared to human cells. In terms of the human cells, some authors have used osteosarcoma cells which are pathologic cell type characterized by aggressive numerous atypical mitoses, and their properties are far different to that of MSCs [59, 62, 69, 71]. Differences could even exist between the same osteoprogenitor cells isolated from humans who suffer from a fracture compared to those who do not, as systemic signals are triggered forcing the cells to proliferate or differentiate toward a specific way as a result of the trauma stimulus sustained [149, 150].

It merits saying that NSAIDs can affect the osteoprogenitor cells by a pathway far different to that of the inhibition of COX-1 and COX-2 enzymes, therefore, differences in tissue culture parameters could influence the final result. This so-called “non-Cox effect” is unfortunately poorly understood. According to this theory, the NSAIDs properties related the protective effect against the tumors, cancer inhibition, and the prevention of metastasis as well as the prevention of other pathologies like Alzheimer's disease or cataract cannot be explained solely by the inhibition of prostaglandins, therefore, an alternative unrelated to COX enzymes inhibition pathway should exist to explain these results [17]. This theory can be strengthened by the studies of Chang et al., who presented that the replenishment of PGE-1, PGE-2, and PGF2a did not reverse this negative effect on bone cells produced by the studied NSAIDs [64, 68].

This comprehensive review includes the data of 18, mainly retrospective, clinical studies trying to enlighten this area of high interest [128–145]. Two early studies involved patients suffering from Colles' fracture reported no effect of NSAIDs on union rates [136, 137]. It is of note, however, that nonunion after Colles' fracture is rare and only a few case reports exist [148]. There are a few case studies that tried to define an association between NSAIDs exposure and nonunion [140–142, 144]. Most of them report an increased incidence of nonunion among the patients that received NSAIDs. Although this can be true, many covariates that could influence this result like smoking, extent of trauma, comminution, patient demographics, and diabetes were not isolated. Unavailable bias of variable forms could exist in a large number of these studies. In a retrospective review of approximately 10,000 humeral shaft fractures treated nonoperatively, the authors reported increased incidence of nonunion among patients receiving NSAIDs [144]. However, opioids user had an increased risk as well. To our knowledge, we are not aware of any studies highlighting a similar effect. So, does this mean that NSAIDs increase the risk for a nonunion or that patients with unhealed fractures require significant amounts of analgesia for prolonged period of time? Another study showed that NSAIDs users had a relative risk of 2.02 (*P* = 0.035) for reoperation compared to nonusers [141]. Similar to the previous example, patients with complications requiring a second operation would have increased analgesic needs. In the same study, the risk for patients receiving antibiotics was 3.01 (*P* = 0.002). Although some antibiotics can interfere with the bone healing process [16], it looks more plausible that the extent of injury and/or a contaminated open fracture is the cause for this observation. These two examples highlight the difficulties faced by the researchers on these observational studies and the potential presence of bias.

In spinal fusion, the results presented are more uniform. Some studies suggest that NSAIDs have no effect on union rates, [130, 132–135] while some others showed a dose-dependant inhibitory effect underlining that patients who continued to take NSAIDs for more than 3 months postoperatively showed significantly lower success rates [128]. Potential presence of bias can exist in these studies as well and it is unclear whether COX-2 selective NSAIDs do have an effect as the vast majority of these studies utilize ketorolac which is a high COX-1 inhibitor. It is worth mentioning that there are significant differences in the nonunion rates between long bones and spine. The nonunion in spinal fusion can be as high as 15% [151]. Differences in structure, mechanical loading, and function of vertebrae also exist. This can result in different degrees of affection, if any, by NSAIDs.

The effect of NSAIDs on heterotopic ossification (HO) has being used as an argument against NSAIDs administration during fracture healing. HO is defined as the process by which marrow-containing boneis formedin soft tissues outside the skeleton [152, 153]. NSAIDs have proven to have a strong effect on this process although the pathophysiology is not fully understood [15]. A Cochraine meta-analysis of 17 trials involving more than 1900 patients having hip joint replacemnet suggested that NSAID administration reduces HO by 59% [152]. Although this is true, HO should not be confused with bone healing as it is a pathologic condition in which a fully differentiated and comitted cell turns into bone. Contributing factors for the development of HO have been proposed to be the locally released BMPs, inflammation and PGE-2 production, hypercalcemia, hypoxia, abnormal nerve activities, immobilization, and disequilibrium of hormones [153, 154]. HO does not follow the fracture healing cascade and significant differences of the local microenviroment characteristics exist as, for example, the mechanical loads that are applied on these tissues are minimal compared to those between the fragments of a broken bone. It is also possible that the fully committed cells switch from one type to another similar to what occurs in the transdifferentiation of MSCs [155].

NSAIDs, due to their ability to inhibit the production of prostaglandins, alleviate the intrinsic local inflammatory response, desensitizing the peripheral pain receptors. Although they are potent analgesics, some studies showed that they can inhibit bone healing, while some others disagreed with these findings. This has triggered a wide range of responses from the medical community, ranging from recommendation of cautious use to statements like “when fracture healing or spine fusion is desired, nonsteroidal anti-inflammatory drugs should be avoided” [156]. The outcome is a widespread confusion with some centres having ignored these recommendations while others rely on narcotic analgesia for pain control even for nondisplaced fractures.

 In the absence of robust scientific evidence concerning the use of NSAIDs after fracture, a definite statement regarding their use cannot be made. However, based on the available literature, some simple carefully derived recommendations can be issued. According to the authors' opinion, there is rather weak evidence to absolute contraindicate the use of NSAIDs in patients suffering from a fracture. NSAIDs administration should be considered as a risk factor for delayed fracture healing, at extreme as equal to smoking, corticosteroids, or diabetes. Clinicians could consider them as low-risk patients and for ashortperiod, probably not exceeding a week after fracture. The need of prospective well-controlled clinical trials is warranted. Patient selection and recruitment, the randomization of patients, and ways to overcome ethical issues are all crucial. In addition, defining the pathway by which NSAIDs could affect bone cells would be of paramount importance. 

## 5. Conclusion

There is no robust clinical and/or scientific evidence to discard the use of NSAIDs in patients suffering from a fracture, but equal lack of evidence does not constitute proof of the absence of an effect. The majority of the available evidence is based on animal findings and these results should be interpreted with caution due to the differences in physiological mechanisms between humans and animals. The need of basic science research defining the exact mechanism that NSAIDs could interfere with bone cells and the conduction of well-randomized prospective clinical trial are warranted. Till then, clinician should treat NSAIDs as a risk factor for bone healing impairment and should be avoided in high-risk patients.

## Figures and Tables

**Table 1:** *In-Vitro* Studies BM: Bone marrow, TB: Trabecular Bone.

Year/Study	Model used	Drug	Outcome
Törnkvist et al., 1984 [56]	Chicken mesenchymal limb-bud cells	Indomethacin (25–100 *μ*M)	(i) No effect on osteogenesis and chondrogenesis

Ho et al., 1999 [57]	Osteoblasts derived from fetal rat calvaria	Ketorolac (0.1–1000 *μ*M), Indomethacin (0.01–100 *μ*M)	(i) All concentration of Ketorolac inhibited proliferation at 24 hours (ii) 0.1 *μ*M of indomethacin or higher inhibited proliferation (iii) A dose dependant increase of ALP was found for concentration between 0.1–100 *μ*M of Ketorolac (iv) Both NSAIDs stimulated collagen type I synthesis

Evans and Butcher,2004 [58]	Human trabecular bone osteoblasts	Indomethacin (0.003–0.3 *μ*M/L)	(i) Inhibition of proliferation and increase in collagen synthesis and ALP in a dose dependant manner

Wang et al., 2004 [59]	MG63 human osteoblasts	Celecoxib (1–120 *μ*M)	(i) Dose dependant decrease of cellular proliferation and stimulation of Ca^++^ production

Chang et al., 2005 [60]	Osteoblasts derived from fetal rat calvaria	Diclofenac, piroxicam, indomethacin Ketorolac (0.001–0.1 *μ*M)	(i) All NSAIDs resulted in cell cycle arrest and cell death (ii) Piroxicam had the least effect to produce osteoblastic dysfunction

Wang et al., 2006 [61]	BM-derived Rat MSCs	Aspirin 1, 5, 10 mmol/L	(i) Inhibition of MSCs proliferation

Wiontzek et al., 2006 [62]	MG63 human osteoblasts	Celecoxib (10 *μ*M)	(i) No effect on Ca^++^ production, COX-2 expression, ALP and osteocalcin

Wolfesberger et al., 2006 [63]	Canine Osteosarcoma cell line	Meloxicam (1–200 *μ*g/mL)	(i) Marked untiproliferative effect for concentrations over 100 while lower concentrations resulted in an increase of cell numbers

Chang et al., 2007 [64]	Human MSCs and D1-cells (Mice)	Indomethacin (10, 100 *μ*M), Celecoxib (1, 10 *μ*M)	(i) Inhibition of proliferation for both NSAIDs but no significant cytotoxic effect (ii) Replenishment of PGE-1, PGE-2 and PGF2a did not reverse this negative effect

Kellinsalmi et al., 2007 [65]	Human MSCs	Indomethacin (1, 10, 100 *μ*M), Parecoxib (1, 10, 100 *μ*M), NS398 (0.03, 0.3, 3 *μ*M)	(i) All studied NSAIDs inhibited osteoblastic and osteoclastic differentiation (ii) Significant increase of adipocytes suggesting diversion to adipogenesis instead of osteogenesis

Arpornmaeklong et al., 2008 [66]	Mouse calvaria cell line MC3T3-E1	Indomethacin (0.1 *μ*M), Celecoxib (1.5, 3, 9 *μ*M)	(i) Inhibition of growth with both NSAIDs (ii) Indomethacin had a higher inhibitory effect than Celecoxib

Abukawa et al., 2009 [67]	Porcine BM progenitor cells	Ibuprofen (0.1, 1, 3 mmol/L)	(i) 0.1 mmol/L had no effect on proliferation, ALP, bone matrix mineralization while inhibition found for the higher studied concentrations

Chang et al., 2009 [68]	Human osteoblasts	Indomethacin (0.1–1 *μ*M), Ketorolac (0.1–1 *μ*M), Piroxicam (0.1–1 *μ*M), Diclofenac (0.1–1 *μ*M), Celecoxib (1–10 *μ*M)	(i) Inhibition of proliferation occurred with all studied NSAIDs (ii) Replenishment of PGE-1, PGE-2 and PGF2a did not reverse this negative effect

Kolar et al., 2009 [69]	MG63 human osteoblasts	Celecoxib (2, 10, 50 *μ*M)	(i) Marginal effect with the concentrations of 2 and 10 *μ*M but 50 *μ*M reduced cell viability and OPG secretion and stimulated oxygen consumption and GLUT-1 expression

Yoon et al., 2010 [70]	Human BM MSCs	Celecoxib (10, 20, 40 *μ*M ), Naproxen (100, 200, 300 *μ*M)	(i) No effect on ALP and Calcium content in absence of Interleukin 1*β* while in its presence ALP and Calcium was reduced only with the highest studied concentration

Guez et al., 2011 [71]	Human MG-63 Osteosarcoma Cell	Indomethacin (1–10 *μ*M) Nimesulide (1–10 *μ*M) Diclofenac (1–10 *μ*M)	(i) All NSAIDs had an inhibiting effect on osteoblastic proliferation and significant effects on the antigenic profile (ii) No treatment altered osteocalcin synthesis

Müller et al., 2011 [72]	Equine BM MSCs	Flunixin (10–1000 *μ*M), phenylbutazone (10–1000 *μ*M), Meloxicam (0.01–200 *μ*M), Celecoxib (0.01–200 *μ*M)	(i) Low NSAIDs concentrations had positive effect on proliferation while the higher ones inhibited proliferation (ii) Adipogenic and chondrogenic differentiation was found unaltered however osteogenesis was significantly disrupted

Pountos et al., 2011 [73]	BM and TB derived MSCs	Diclofenac, Ketorolac, Parecoxib, Ketoprofen, Piroxicam, Meloxicam and Lornoxicam (all 0.001 to 100 *μ*g/mL)	(i) No effect on MSCs proliferation when cellular medium was supplemented with expected plasma concentrations (ii) Negative effect encountered when high concentrations used (over 100 *μ*g/mL) (iii) NSAIDs in plasma concentrations had no effect on osteogenesis (iv) Chondrogenesis was found inhibited by NSAIDs

**Table 2:** Animal studies: agents and model used in relation to the presented effect.

Impaired bone healing	
(1) Aspirin [84]	
(2) Celecoxib [101, 102]	
(3) Diclofenac [97–99]	
(4) Etodolac [104, 105]	
(5) Ibuprofen [88, 94–96, 127]	
(6) Indomethacin [78–91, 119, 127]	
(7) Ketoprofen [77]	
(8) Ketorolac [74–76, 107]	
(9) Meloxicam [85, 103]	
(10) Naproxen [92, 93]	
(11) Parecoxib [74]	
(12) Rofecoxib [92, 94, 95, 108, 109]	
(13) Tenoxicam [99, 100]	
(14) Valdecoxib [107]	

No effect	
(1) Celecoxib [80, 111, 119, 120]	
(2) Diclofenac [123]	
(3) Etoricoxib [110]	
(4) Ibuprofen [111, 121, 122]	
(5) Indomethacin [81, 111, 114–118]	
(6) Ketoprofen [112, 113]	
(7) Ketorolac [110, 111]	
(8) Meloxicam [113]	
(9) Nimesulid [124]	
(10) Rofecoxib [111, 123, 125]	

Short term has no effect	
(1) Diclofenac [98, 106]	
(2) Ketoprofen [126]	
(3) Ketorolac [78]	
(4) Parecoxib [106]	
(5) Rofecoxib [85, 108]	
(6) Valdecoxib [106]	

Model used	
(i) Rats [74, 77, 78, 80, 81, 83, 84, 89–91, 93, 94, 97, 98, 100–107, 110, 114, 116, 118, 121–124, 126]	
(ii) Mouse [109, 111, 120]	
(iii) Rabbit [75, 76, 79, 82, 85–88, 92, 95, 96, 99, 108, 112, 115, 119, 125]	
(iv) Dog [117]	
(v) Goats [113]	

**Table 3:** The effect of NSAIDs on spinal fusion in humans.

Study/Year	Design	NSAID used	Conclusions and recommendations
Deguchi et al., 1998 [128]	Retrospective review of 73 patients undergoing primary or revision one or two level lumbar fusion	Not specified	(i) Patients who continued to take NSAIDs for more than 3 months postoperatively showed significantly lower fusion and success rates

Glassman et al., 1998 [129]	Retrospective review of 288 patients undergoing posterior L4 to sacral fusion	Ketorolac	(i) High rate of nonunion in spinal fusion (ii) Avoid NSAIDs in early postoperative period is recommended

Vitale et al., 2003 [130]	Retrospective review of 208 children undergoing scoliosis correction	Ketorolac	(i) No significantly increase in complications, including transfusion and reoperation

Park et al., 2005 [131]	Retrospective review of 88 consecutive patients undergoing posterolateral lumbar fusion	Ketorolac	(i) The incidence of incomplete union or nonunion was much higher in the ketorolac group, and the relative risk was approximately 6 times higher than control group

Pradhan et al., 2008 [132]	Retrospective review of 405 consecutive patients undergoing one, two or three level posterolateral lumbar fusion	Ketorolac	(i) The use of ketorolac limited to 48 hours after surgery for adjunctive analgesia, has no significant effect on ultimate fusion rates.

Sucato et al., 2008 [133]	Retrospective review of 319 patients undergoing scoliosis correction	Ketorolac	(i) Ketorolac does not increase the incidence of developing a pseudoarthrosis when used as an adjunct for postoperative analgesia

Lumawig et al., 2009 [134]	Retrospective review of 273 patients undergoing one or two level posterior lumbar fusion	Diclofenac	(i) Diclofenac sodium showed a dose-dependent inhibitory effect toward spinal fusion especially when used during the immediate postoperative period

Horn et al., 2010 [135]	Retrospective review of 46 pediatric patients who undergone spinal fusions for scoliosis	Ketorolac	(i) No clinical or radiographic evidence of curve progression, nonunion, or instrumentation failure

**Table 4:** Studies analyzing the effect of NSAIDs on bone healing in humans.

Study/Year	Design	NSAID used	Conclusions and recommendations
Davis and Ackroyd, 1988 [136]	Prospective double-blinded study of 100 patients with Colles' fracture	Fluriprophen (50 mg TDS)	(i) No effect on Colles' fracture.

Adolphson et al., 1993 [137]	Randomized double-blinded study on 42 postmenopausal women with colles fracture	Piroxicam	(i) No decrease of the rate of fracture healing (ii) Patients receiving piroxicam had significantly less pain (iii) No difference in the rate of functional recovery

Butcher and Marsh, 1996 [138]	Retrospective review of 94 patients with tibial fracture	Not specified	(i) Increase in the length of time to union by of 7.6 weeks (*P* = 0.0003) (16.7 weeks versus 24.3 weeks).

Wurnig et al., 1999 [139]	80 prospective patients receiving indomethacin prophylaxis for THR compared with 82 patients without	Indomethacin (Oral 50 mg BD)	(i) No effect on prosthetic loosening after cementless hip arthroplasty

Giannoudis et al., 2000 [140]	Retrospective review of 377 patients treated with IM nail	Ibuprophen and Diclofenac	(i) Increased risk for nonunion in patients receiving NSAIDs

Bhandari et al., 2003 [141]	Retrospective review of 192 tibial shaft fractures	Not specified	(i) Relative risk of 2.02 (*P* = 0.035) for patient who take NSAIDs

Burd et al., 2003 [142]	Retrospective review of 282 with acetabular fractures	Indomethacin	(i) Patients receiving indomethacin had increased risk for developing non-union

Sculean et al., 2003 [143]	Randomized blindied study on 20 patients with deep intrabony defect	Rofecoxib (25 mg/day for 14 days)	(i) No effect on the healing of intrabony periodontal defects

Bhattacharyya et al., 2005 [144]	Retrospective review of 9995 humeral shaft fractures treated nonoperatively	Not specified	(i) Exposure to nonselective NSAIDs in the period 61–90 days after a humeral shaft fracture was associated with nonunion

Meunier et al., 2009 [145]	Randomized study involving 50 patients undergoing total knee replacement	Celecoxib (200 mg BD)	(i) No differences in prosthesis migration, pain scores, range of motion, and subjective outcome were found after 2 years
